# Green Chemistry Extractions of Carotenoids from *Daucus carota* L.—Supercritical Carbon Dioxide and Enzyme-Assisted Methods

**DOI:** 10.3390/molecules24234339

**Published:** 2019-11-27

**Authors:** Natalia Miękus, Aamir Iqbal, Krystian Marszałek, Czesław Puchalski, Artur Świergiel

**Affiliations:** 1 Department of Animal and Human Physiology, Faculty of Biology, University of Gdańsk, Wita Stwosza 59, 80-308 Gdańsk, Poland; miekusn@gmail.com (N.M.); swiergiel@yahoo.com (A.Ś.); 2 Department of Pharmaceutical Chemistry, Medical University of Gdańsk, Hallera 107, 80-416 Gdańsk, Poland; 3 College of Food Science and Technology, Huazhong Agricultural University, Wuhan 430070, China; aamirraoiqbal@yahoo.com; 4 Department of Fruit and Vegetable Product Technology, Prof. Wacław Dąbrowski Institute of Agricultural and Food Biotechnology, 36 Rakowiecka, 02-532 Warsaw, Poland; 5 Department of Chemistry and Food Toxicology, Institute of Food Technology and Nutrition, College of Natural Science, University of Rzeszow, Ćwiklińskiej 2D, 35-601 Rzeszow, Poland; 6 Department of Bioenergetics and Food Analysis, Faculty of Bogy and Agriculture, University of Rzeszow, Ćwiklińskiej 2D, 35-601 Rzeszow, Poland; cpuchal@ur.edu.pl; 7 Prof. Wacław Dąbrowski Institute of Agricultural and Food Biotechnology, 36 Rakowiecka, 02-532 Warsaw, Poland

**Keywords:** carrot, carotenoids, extraction, green chemistry, supercritical carbon dioxide

## Abstract

Multiple reviews have been published on various aspects of carotenoid extraction. Nevertheless, none of them focused on the discussion of recent green chemistry extraction protocols, especially for the carotenoids extraction from *Daucus carota* L. This group of bioactive compounds has been chosen for this review since most of the scientific papers proved their antioxidant properties relevant for inflammation, stress-related disorders, cancer, or neurological and neurodegenerative diseases, such as stroke and Alzheimer’s Disease. Besides, carrots constitute one of the most popular sources of carotenoids. In the presented review emphasis has been placed on the supercritical carbon dioxide and enzyme-assisted extraction techniques for the relevant tetraterpenoids. The detailed descriptions of these methods, as well as practical examples, are provided. In addition, the pros and cons of each method and comparison with the standard solvent extraction have been discussed.

## 1. Introduction

**Figure 1:**
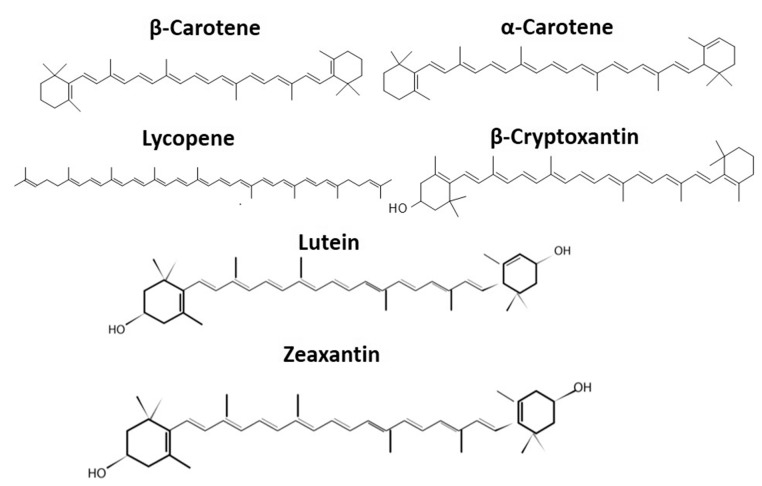
Chemical structures of selected carotenoids.

Carotenoids belong to the isoprenoid group of pigments that are produced by both photosynthetic plants and some non-photosynthetic fungi and bacteria. Most animals cannot synthesize carotenoids and have to obtain them from foods [1]. Carotenoids are important phytochemicals and have been studied extensively for their health benefits. Moreover, they are valuable to the food industry because they can be used as natural food colorants to provide a range of pigments, from yellow to red. Food color also has a huge impact on consumer perception of quality [2,3]. There are over 600 known carotenoids, mostly existing in two structural forms: polyunsaturated hydrocarbons and oxygenated hydrocarbons, commonly labeled as carotenes and xanthophylls, respectively [4,5] (Figure 1). Both xanthophylls and carotenes provide color to biological materials and are valuable for the nutraceutical market, but they differ in structures and activities. Carotenoids are also classified into two categories: pro-vitamin A carotenoids that can be converted into retinol, e.g., mutatochrome, β-carotene, and β–cryptoxanthin, and non–provitamin A carotenoids that are unable to convert into retinal carotenoids such as lutein and lycopene [6]. β-carotene has pro-vitamin A properties. Vitamin A is biologically relevant mainly due to its antioxidant properties. It protects the body from free radical cell damage that could trigger the growth and replication of abnormal cells resulting in cancerous tumors. Also, the deficiency of vitamin A has a huge impact on immunity and could lead to the damage of light-sensitive receptors [7,8]. β-carotene is cleaved in half by the enzyme carotene deoxygenase, thus becoming a molecule displaying vitamin A activity [9]. Other xanthophyll carotenoids such as β-cryptoxanthin and lutein/zeaxanthin are beneficial for bone and eye health, respectively. Both of these xanthophylls are the only carotenoids found in the macular of the retina [10]. Consequently, they have been studied extensively for their ability to lower the occurrence of cataracts and macular degeneration in the human eye [11,12]. In addition to these beneficial effects, carotenoids also play an important role in cardiovascular health and cognitive functions [2,12].

Due to various disadvantages inherent in carotenoids extraction by conventional organic solvents, new green extraction techniques, such as supercritical fluid extraction and enzyme-assisted extraction has been investigated for extracting these bioactive compounds [13]. These technologies have been widely used to extract carotenoids from a range of fruit and vegetable matrices, such as carrot, tomato watermelon, pumpkin [14,15,16], and also from fruit and vegetable waste matrices including banana, grape and tomato peels, grape, pomegranate and pumpkin seeds, as well as apricot bagasse and pomace [17,18,19,20]. The obtained extracts were evaluated for their carotenoid contents, antioxidant activity, and carotenoid recovery. The results showed that the new green extraction techniques for carotenoid extraction are more suitable to generate carotenoid-rich extracts [21,22]. Carrots are the second richest source of β-carotene after dehydrated pepper, while grape leaves are considered to be the third-largest source of β-carotene [23]. Carrots generally contain carotene in the range of 60–120 mg/100 g, while some varieties of carrots contain higher amounts of carotene (300 mg/100 g). In carrots, 80% of carotene found consists of β-carotene bounded to proteins [24,25]. This β-carotene in carrots provides more amount of absorbed vitamin A compared to other vegetables that have an inferior absorption of β-carotene [26]. Fresh leaves of spinach, dandelion green, turnip green, and sweet potato are considered to be rich sources of zeaxanthin and lutein, while lycopene is present abundantly in tomato fruit [27]. Some studies also suggest that other fruits, such as bitter melon (*Momordica charantia* L.) also bear high contents of lycopene [6,28]. Green leaves of softwood-tree from *Moringaceae* family (*Moringa oleifera*) have been demonstrated to contain a high content of carotenoids such as β-carotene and lutein. Similarly, *Dunaliella salina* and *Dunaliella bardawil* (unicellular microalgae, Chlorophyta) are also rich sources of β-carotene [3].

## 2. Chemical Properties of Carotenoids Important for Their Isolation

Carotenes consist of long polyunsaturated hydrocarbon chains, making them nonpolar (Figure 1). They are soluble in organic solvents such as petroleum ether and hexane [10]. Xanthophylls are oxygenated carotenoids, making them more polar than carotenes [29]. They are soluble in semi-polar solvents such as ethanol and methanol. This type of carotenoid has no pro-vitamin A activity because of the hydroxyl groups present on either one or both ends of the xanthophyll structure. Lutein and zeaxanthin are two specific types of xanthophylls. β-cryptoxanthinhas hydroxyl derivatives, canthaxanthin have keto derivatives, while violaxanthin and beta-citraurin have epoxy and aldehyde derivatives respectively [25].

Figure 1 shows some carotenoids that could be acyclic (such as lycopene) or di-cyclic [30]. Alpha-carotene (α-carotene) and beta-carotene (β-carotene) are di-cyclic. In nature, carotenoids mostly exist in a stable all-*trans* form, while only small quantities may be present in *cis*-conformers [25,30]. In addition to these two categories, apocarotenoids and C50 carotenoids comprise other classes of carotenoids. Apocarotenoids are formed by the oxidative cleavage of carotenoids by a catalyst dioxygenase. Several apocarotenoids such as bixin pigment, abscisic acid, α-ionone, and β-ionone may be of biological and commercial importance [31]. Many soil bacteria such as *Corynebacterium glutamicum* have a significant role in the production of C50 decaprenoxanthin that are potentially utilized in cosmetic production, due to having UV light-protecting properties [6,32].

Carotenoids are lipophilic and have a highly conjugated system that makes them prone to isomerism and oxidation [25]. During isomerization, carotenoids change their isomerism from a stable *trans*-form to an unstable *cis*-form. On oxidation, carotenoids may form different low molecular weight products, such as epoxycarotenoids, apocarotenoids or hydroxycarotenoids, depending on the parent carotenoid [33]. Carotenoid degradation pathways are highly influenced by the agents involved in the oxidation initiation. Once oxidation is initiated by one of the oxidizing agents, carotenoid may further react with themselves or other chemical species within their environment to form a plethora of products [34]. Heat, light, acids, and adsorption on an active surface, such as alumina, promote isomerism of *trans*-carotenoid to *cis*-carotenoids. This leads to a loss of color and anti-oxidant activities [35]. Thus, oxidative degradation is the principal cause of extensive losses of carotenoids. Degradation occurs with the availability of oxygen and is stimulated by light, enzymes, metals, and co-oxidation with lipids hydroperoxide [36].

**Figure 2:**
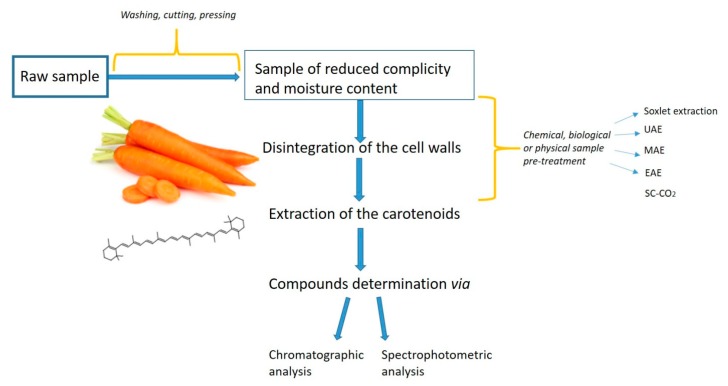
Main steps in carotenoids extraction—flowline.

For efficient extraction of carotenoids from natural sources, a simple, rapid, and inexpensive extraction method is the prime requirement (Figure 2). The quantitative information derived from unbiased extraction and determination procedures may be useful in assessing carotenoids biosynthesis and accumulation [25]. One concern with all carotenoids (xanthophylls and carotenes) is that the conjugated double bonds in their structure make them susceptible to oxidation in the presence of heat, light, unsaturated fats, peroxides, and some metals [37]. Additionally, heat, light, acids, and refluxing in an organic solvent can cause the carotenoids to isomerize from the natural *trans*-state to the *cis*-state resulting in reduced color intensity and Vitamin A activity [38]. Carotenoids degradation is an important aspect to consider when developing an extraction method and aiming at maximizing said extraction.

The green chemistry approaches in laboratories are being implemented in various areas. Among the preferable methods for green chemistry, those allowing for the reduction of the amount of environmentally hazardous organic solvents are of great relevance. These methods are relatively cheap, fast, and environmentally safe, and could be applied for clinical and food analysis, to name a few [38,39]. Nardi et al. [40] developed many green extraction techniques and accomplished a complex protocol of natural phenolic extraction from extra virgin olive oil by using some nonconventional methods of demethylation and deglycosylation mediated by enzymes. The resultant phenolic was considered as natural food additive compounds, as well as protective agents against lipid peroxidation in biological systems [40]. Similarly, some phenolic compounds such as oleuropein aglycone derivatives were also synthesized by transacetylation with different fatty alcohols in the presence of Lewis acid catalyst under mild conditions with the aim of making the oleuropein suitable for food fats [41]. Especially, while analyzing health beneficial compounds from plants, such as carotenoids, the application of those “green” methods should be strongly recommended. Multiple analytical methods were evaluated for the carotenoid’s extraction from natural matrices. Among them is the extraction with Soxhlet, maceration, microwave, or ultrasound-assisted extractions, or the supercritical fluid extraction (commonly based on the use of supercritical carbon dioxide (SC-CO_2_) and enzyme-assisted extraction) [6,42,43,44]. On the one hand, microwave- or ultrasound-assisted extraction allowed the plants’ cell wall disruption, thus improving the extraction [45]. Nevertheless, these methods require high amounts of energy and result in only mediocre efficiency. On the other hand, supercritical carbon dioxide and enzyme-assisted extractions (SC-CO_2_ and EAE, respectively) are two of the extraction methods that fulfill the green chemistry postulates. They are believed to be environmentally friendly, have shorter extraction times, higher extraction yields, and better retention of nutritional and valuable bioactive compounds, while compared to traditional solvent or mechanical pressing approaches (Table 1) [46,47]. Also, scientific data revealed that by applying the SC-CO_2_-based methods enzyme and microbial inactivation was achieved for apples or orange juices, among others [48,49,50]. This review aims at presenting and the critical review of some SC-CO_2_ and EAE-based methods for carotenoids extraction from *Daucus carota L*. described in papers published between 2009 and 2019.

### 2.1. Aspects to Consider: Carotenoids Extraction

Due to a difference in the genotype and structure of food samples, there are no commonly established carotenoids extraction procedures or standard methods [37,51]. However, most of the extraction methods involve the releasing of desired carotenoid components by disrupting tissues of food matrices, followed by removing undesirable components and a liquid–solid or liquid–liquid extraction [51]. The selection of solvents to extract carotenoids depends mainly on the polarity of existing carotenoids and the food matrix structure and components. Mostly, non-polar solvents, such as hexane, prove more appropriate for extraction of non-polar or esterified carotenoids (carotenes), while polar solvents, such as acetone and ethanol, are a good choice for polar carotenoids (xanthophylls). Furthermore, the susceptibility of carotenoids to oxidation should be considered during the development of a superior extraction method, as carotenoids are very sensitive to light, heat, acid, or oxygen exposure. The various steps involved in the general extraction of carotenoids are summarized in Table 2.

The high water content of food sources is considered a negative factor for an efficient carotenoid extraction, particularly when considering superfluid extraction (SFE), because of the hydrophobic nature of solvents and carotenoids [6,56]. Thermal-based extraction methods such as heating, oven or microwave drying could cause heat degradation of carotenoids [57,58]. Therefore, food samples are dehydrated using a lyophilizer to protect carotenoids from thermal degradation. However, this procedure could increase the time and cost of carotenoids extraction. Moreover, carotenoids may be subjected to degradation even at low temperatures during the cellular disruption of food samples necessary for the process, while carotenoids isolation is needed.

In general, a few points should be followed to reduce the degradation processes during carotenoids extraction: (i) a carbonate-based neutralizers such as sodium bicarbonate, calcium carbonate, or magnesium carbonate should be added to neutralize acids generated from plant samples, as the acids can hinder the extraction of carotenoids; (ii) antioxidants such as butylated hydroxytoluene, tert-butlylhydroqinone, or ascorbyl palmitate can be added to prevent oxidation during carotenoids extraction; (iii) extraction time should be minimized to avoid enzymatic oxidation and efficient extraction of carotenoids; (iv) food samples should be protected from UV light to avoid photodestruction of carotenoids; and (v) sample tubes should be cleaned with nitrogen to remove oxygen and offer an inert environment [6,59].

### 2.2. Pre-Treatments Applied Before Extraction of Carotenoids

The complex and rigid cell wall present in plant structures could hinder the entry of solvents inside the cells to extract carotenoids. Also, the linkages between carotenoids and other macromolecules (proteins and fatty acids) could further reduce the efficiency of carotenoids extraction. Thus, during the extraction of relevant phytochemicals, various sample pre-treatment methods are applied. Their main objective is the breakdown of the cell wall and other physical barriers in the food samples, thus permitting an efficient carotenoids extraction [60]. Among those methods: physical, enzymatic, biological, and chemical, some pre-treatment approaches could be utilized. Physical pre-treatment methods include drying, freeze-thaw cycles, cooking, and cryogenic grinding, while chemical methods are based on the application of acid, base or surfactants. These methods are employed wisely, based on the characteristics of cell wall and cellular matrix. For instance, intense pre-treatment methods are required to break the robust tri-layered cell wall in *Haematococcus lacustris* (formerly *H. pluvialis,* Chlorophyta) [6]. Mezzomo and Ferreira [61] studied many types of pre-treatment methods and found cooking as one of the best technique to achieve a high yield of carotenoids in pink shrimp (*Pandalus brasiliensis and P. paulensis*) residue, compared to milling and dehydration pre-treatments [61,62]. On the other hand, cryogenic pre-treatment, consisting of precooling, grinding and intermediate cooling was found to be the best method for carotenoids extraction in microalga *Ettlia oleoabundans* (formerly *Neochloris oleoabundans,* Chlorophyta) [63,64]. Higher recoveries of lutein and β-carotene were observed after saponification of cereal than after extraction without saponification [65]. In this study, the authors also monitored the time for saponification and concentration of the alkaline and revealed that those parameters should be adjusted according to the food matrix in order to achieve a maximum carotenoids extraction yield. Amiri-Rigi and Abbasi [66] studied micro-emulsion pre-treatment using different enzymes, surfactants (Tweens, span 20, saponin, sucrose monopalmitate, and lecithin) and co-surfactants (glycerol, 1-propanol, ethanol, and propylene glycol) for lycopene extraction from tomato pomace. The highest extraction of lycopene was obtained by a combination of these pre-treatments [66]. Ultrasound was also found to be an effective pre-treatment for mechanical disruption of cell wall, to secure the utmost extraction yield out of astaxanthin. High-pressure homogenization (HPH) was also useful for cell disruption and improved the recovery of thermolabile compounds such as carotenoids, phenolic acids, flavonoids, lignans, and other polyphenols [67,68]. Many scientists have reviewed different pre-treatment methods of cell wall disruption to get an efficient extraction of carotenoids [69] but different factors, such as cost, energy consumption, time, and metabolite stabilities still need to be investigated.

### 2.3. Selection of the Most Appropriate Solvent

Solvent extraction is the most widely used method due to its simplicity and having been scaled up industrially in the past. Different organic solvents such as chloroform, hexane, methanol, diethyl ether, acetone, isopropanol, and methylene chloride have been used to efficiently extract carotenoids. Moreover, a combination of these solvents has been utilized to get synergistic effects on carotenoids extraction. The selection of appropriate solvent or combination of said solvents depends on the polarity and chain length of the target carotenoids but the food matrix components and moisture contents also play an important role in solvent selection [37]. Mostly, hexane and acetone are used for the extraction of non-polar and polar carotenoids, respectively. On the other hand, mixtures of different solvents such as acetone, ethanol or hexane are utilized for the simultaneous extraction of nonpolar and polar carotenoids. Acetone and ethanol have been used to extract carotenoids from highly moisturized food materials due to the water-miscible properties of these solvents [51]. Those two solvents are also preferred over the solvents such as hexane, diethyl ether, dichloromethane, and chloroform since they have less environmental, health and safety impact [12].

Still, solvent extraction requires large amounts of organic solvents and can also cause the degradation of carotenoids when heating is applied (a process necessary for some of the solvents). Thus, another method was tested for carotenoid extraction. One of them is solid-phase extraction (SPE). SPE uses solvents and a solid media to separate desired components from a liquid matrix. This method uses smaller volumes of solvents than standard solvent extraction and could have the selectivity to separate very similar compounds from each other [32]. SPE is still not considered a perfectly “green approach”. Therefore, supercritical fluid extraction was developed. It takes advantage of the unique properties that materials possess in supercritical states, such as high diffusivity, increased density, and low viscosity. Some supercritical fluids, such as carbon dioxide or propane, are strong solvents when they are compressed and heated. Supercritical extraction is advantageous because it minimizes the use of organic solvents. Therefore, more green solvents and environmentally friendly liquids might be explored for the extraction of bioactive compounds and carotenoids from biological matrices.

## 3. Supercritical Carbon Dioxide (SC-CO_2_) Extraction

**Figure 3:**
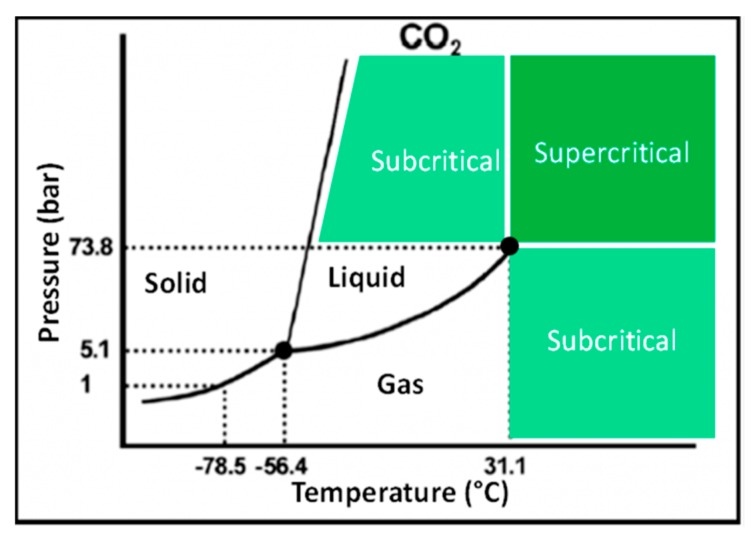
A typical phase diagram indicating the different states of matter at given pressure and temperature settings [75].

SC-CO_2_ is a gas or liquid that has been compressed and heated beyond a critical pressure and temperature [70,71]. At the supercritical phase, CO_2_ possesses liquid-like density and has intermediate physiochemical properties between liquids and gases (Figure 3). CO_2_ critical point is found at 31.1 °C (304.2 °K) and 7.3 MPa (72.8 bar), allowing to operate near room temperature and mild pressure [72], which is ideal to extract the thermo-labile and (oxidizable) natural food components such as carotenoids and phenolic compounds. The higher diffusion and lower viscosity of CO_2_ at supercritical state could lead to rapid penetration of CO_2_ into the pores of complex food matrices, thereby, enhancing the efficiency of carotenoids extraction [61]. Additionally, the obtained carotenoid extracts are highly concentrated, leaving no toxic organic solvents in the final product [73,74].

SC-CO_2_ is a non-polar solvent that can replace one of the most commonly used non-polar solvents such as hexane. The compound’s solubility in SC-CO_2_ is dependent on the compound’s polarity, molecular weight, and structure [76]. Components with lower molecular weight and lower polarity could be extracted easily at low pressures with SC-CO_2_ because they could perfectly match the solvent’s polarity at this pressure. Moderate to highly polar compounds are almost insoluble in SC-CO_2_ [77]. The pressure and temperature of the fluid can be adjusted to better solvate certain compounds. Sometimes, adjusting these parameters is not enough and a solvent modifier needs to be added during extraction to adjust the overall polarity of the solvents. Methanol and ethanol are often used as solvent modifiers to increase the extraction of polar compounds [51]. SC-CO_2_ extraction, especially when coupled with a modifying solvent, can be successfully used as an extraction solvent. Extracts obtained with SC-CO_2_ are widely used in food applications because carbon dioxide is inert, non-flammable and inexpensive. Furthermore, the extracts obtained with SC-CO_2_ are odorless, tasteless and “generally recognized as safe” (GRAS), because the commercial CO_2_ gas stream can be recycled, and SC-CO_2_ extraction is regarded as a green extraction (environmentally friendly) process [70,78]. Singh, Ahmad, et al. [79] have reviewed different conventional and non-conventional methods of carotenoid extraction, and concluded that SC-CO_2_ under optimized conditions is the best method among others to obtain the optimum extraction yield of environmentally safe, non-toxic and high purity carotenoids [79].

SFE extraction has gained popularity in the last three decades because when carbon dioxide is used as a solvent, the extracts obtained are considered natural and contaminant-free [80]. SC-CO_2_ extraction has also been the subject of much research and development over the years for the extraction of various compounds from samples derived from nature. Though SFE extraction can be performed on various sample types, the basic system for all extractions is the same [77]. The four primary components included in an SFE extraction system are the high-pressure pump, heater, extraction chamber, and separation chamber. The fluid is heated and pressurized before being pumped into the extraction chamber. This chamber is able to withstand extreme pressure conditions. Following extraction, the extract-laden fluid exits the pressurized chamber and undergoes the separation step where a reduction in pressure causes precipitation of the extract. The solvent, free of any extract, can then reenter the pump to be pressurized for reuse. Some systems are equipped with more complex separation chambers, especially if the goal is to separate more than one component in the extract. The separation chamber can be held at different pressures and/or temperatures in order to facilitate the precipitation of only certain components in the extract [77,81,82]. Rapid depressurization during SC-CO_2_ causes cell disruption to remove carotenoids with reduced time and labor requirements [76]. SC-CO_2_ extraction of polar carotenoids (xanthophylls) and non-polar carotenoids (β-carotene) require appropriate levels of temperature, CO_2_ density, and pressure and flow rates. In general, extraction temperature from 40 to 60 °C, pressure from 300 to 400 bar, treatment time from 30 to 120 min, appropriate CO_2_ density, CO_2_ flow rate from 1 to 5 mL/min, and concentration of entrainers from 5 to 25% *v*/*v,* are the five most important parameters during SC-CO_2_ extraction of carotenoids [83,84,85]. The examples of optimized extraction conditions for the carotenoids from different sources are listed in Table 2. In a comparative study, SC-CO_2_ extraction of carotenoids was done by using solvents such as *N*,*N*′-dimethyl formamide and methanol from the microalgae *Dunaliella salina* [86,87]. The study performed by Pour Hosseini, Tavakoli, and Sarrafzadeh, [88] showed that the highest extraction yields of non-polar carotenoids ensued at 60 °C and 400 Ba [88]. Thus, by optimizing different key parameters and organic modifiers (co-solvent) such as ethanol, the efficiency of carotenoids extraction can be significantly enhanced by increasing the solubility of analytes, and by reducing their interaction with the sample matrix which together facilitate the release of said analytes from the sample matrix [89].

### SC-CO_2_ Extraction of Carotenoids from Daucus Carota L.

The total carotenoid contents in carrot vary from 4.6 to 548 μg/g, depending on the different cultivars [90]. The total carotenoids in carrot are composed of β-carotene and α-carotene in the range of 60% and 30%, respectively. Other carotenoids such as lycopene and lutein are present in very lower concentrations [13,90]. The general concentration of different carotenoids in carrots is shown in Table 3. Although SC-CO_2_ without any modifier should efficiently extract from carrots the carotenoids with non-polar nature, a low extraction rate (34%) was achieved due to the high molecular weight of the targeted compounds. Therefore, ethanol as a co-solvent may be used to enhance the recovery rate of targeted compounds. Ethanol has the ability to enhance the polarity of CO_2_, dissolving several polar macronutrients such as proteins, carbohydrates, and lipids. Thus, the high mass yield of extract might be obtained after using a high concentration of ethanol as a co-solvent. In addition to the concentration of the co-solvent, other parameters such as temperature and pressure used during SC-CO_2_ are also important factors that affect the process of carotenoids extraction. Among these factors, pressure plays the main role in increasing the solvation power of CO_2_, enhancing the extraction of carotenoids and other phytochemicals [91]. Moreover, high pressure can also disrupt the cell walls structures and other stronger interactions between different compounds, causing dissociation of carotenoids from complex structures to enhance their extraction [92,93]. High temperatures, up to some extent, can increase carotene extraction but the extreme temperatures can compromise the bioactivity and stability of extracted carotene by causing their degradation and isomerization [91]. Scientists have mostly studied the pressure range between 200 and 450 Ba, and a temperature range between 50–70 °C [18,94]. Some studies reported that the extraction of carotenoids for carrots could generate a lower concentration of carotenoids due to rigid composition and strong interaction among different components (carbohydrates, proteins, lipids, etc.) in carrots. In addition, a high amount of fiber, mostly composed of cellulose and hemicellulose, results in a rigid structure that hinders the carotenoid extraction in carrots [95,96,97]. Therefore, pretreatment with an appropriate amount of co-solvent (pressure and temperature are also important parameters) should be applied to significantly enhance the carotenoids extraction from carrots. The study performed by de Andrade Lima et al. [90] revealed that SC-CO_2_ could extract 96.2% carotenoids from carrot peel when the extraction vessel’s full capacity was used with appropriate temperature and pressure. Also, an application of the SC-CO_2_-based methods together with the enzyme and microbial inactivation for apples or orange juices was shown to be beneficial for carotenoids isolation [48,49,50]. Another study conducted by Kaur et al. (2018) determined the kinetics of the SC-CO_2_ extraction of β-carotene from tray dried carrots at 40, 50, and 55 °C and 30, 35 and 40 MPa (SC-CO_2_ flow rate 2.0 L/min, extraction time up to 6 h) [28]. They pointed out that the mass of crude extract and β-carotene increased with time, temperature and pressure of extraction. The maximum was obtained when extraction was carried out at 45 °C and 35 MPa and 6 h was necessary to reach the equilibrium. Weibull model (Equation (1)) describes adequately the supercritical extraction of β-carotene from carrots.

(1) $$\frac{C-C_{\infty }}{C_{\infty }}=\exp\left(-kt\right)$$

where *C* is concentration of β-carotene in the extract (µg/g) at time *t* = ∞; *k* is extraction rate (h−1); and *t* is the time (h)

**Equation (1).** The Weibull model for the description of the supercritical extraction of β-carotene from carrots.

Besides being used for the carotenoid’s extraction from carrot, SC-CO_2_ with pressure in the range of 10 to 40 MPa could be used for continuous non-thermal pasteurization of carrot juices. It leads to the significantly extended shelf life of the juice, with no loss in most of the nutritional, and functional properties of the raw extracts [108]. Also, SC-CO_2_ was applied as a mild technology to enhance the microbial and enzymatic stability of the product, without altering its main qualitative aspects, for the pasteurization of freshly cut carrots with no modification of their texture, color and nutritional properties [93]. The mentioned studies show the relevance of this method in the nutraceutical and “functional food” markets that demand adequate nutritional quality [109].

## 4. Enzyme-Assisted Extractions (EAE)

As stated above, the solvent-based extractions of bioactive compounds from natural sources often suffer from low extraction yields, require long extraction times and the final product may contain trace amounts of organic solvents that have a huge negative impact on the product quality. The EAE was shown to be effective, environmentally friendly and a selective method for bioactive compound extraction. Enzymes can be perfectly matched catalysts to assist in the extraction of various bioactive compounds from natural origins. The inherent ability possessed by enzymes to catalyze reactions with high specificity, to degrade or disrupt cell walls and membranes and to operate under mild processing conditions in aqueous solutions makes the EAE an interesting alternative for the more efficient extraction of phytochemicals from biological matrices [110]. EAE has a better profile in terms of the disruption of the cell walls, while compared to the microwave- and ultrasound-assisted extraction because a higher efficiency the process is achieved with a lower energy expenditure [46]. Recently, enzyme-assisted technology has been widely used to extract bioactive compounds from many plants. The addition of specific enzymes during the extraction improves the recovery of a compound of interest by breaking the cell wall and hydrolyzing the structural polysaccharides since some compounds are retained in the polysaccharide-lignin network by hydrogen or hydrophobic bonding, and are hardly accessible with a solvent in a routine extraction process [111]. The successful application of enzymes for the extraction of the carotenoids, vanillin, polysaccharide, oil, and polyphenols, among others, was shown many years ago and is still actively investigated [112,113,114,115].

Among enzymes that could be applied in the EAE approach various enzymes, especially highly complex large polymers such as proteases, pectinase, cellulase, tannase, or carbohydrases could be mentioned [116]. In 2010 Wang et al. [117] presented the extraction method of phenolic compounds from *Palmaria palmata (red algae, Rhodophyta)* with the use of proteases and carbohydrases, whereas Fernández, Vega and Aspé [118] used pectinase, cellulase, and tannase enzymes for the extraction of phenolic compounds from the skins and seeds of grape with pectinase being the most effective for the extraction efficiency of phenolic compounds [117,118]. Other, well-studied, bioactive compounds are volatile compounds such as those found in garlic. EAE with the use of cellulase, pectinase, protease, individually, as well as a commercial mixture of enzymes—Viscozyme^®^ L (consisting of cellulase, hemicellulase, arabinase, xylanase, amylase, and β-glucanase, St. Louis, MO, USA) might be used for the pretreatment of garlic. Enzyme pretreatment of garlic resulted in a higher yield of oil (0.5%), compared to the control (0.28%), without any meaningful differences in physicochemical properties of the volatile oil [119]. The water uses a solvent instead of organic chemicals in EAE for the extraction of bioactive compounds makes this technique eco-friendly and perfectly fulfills the “green chemistry” postulates [111].

### 4.1. EAE of Carotenoids

There are numerous reports on the extraction of carotenoids from vegetable sources, with the preferred source being tomato peels and carrots. But other plant matrices are also under investigation. Nath et al. [23] presented the application of three carbohydrases enzymes: Viscozyme^®^ L, pectinase and cellulase for the liquefaction ability in terms of recovery of total carotenoids, total phenolics, total flavonoids and ascorbic acid from the red capsicum extract. Obtained extracts were planned to be used as natural colorants and functional ingredients in foods. The authors studied the performance of the above-mentioned enzymes and their utility for carotenoid extraction. Viscozyme^®^ L and pectinase were observed to cause significantly higher liquefaction that was proved by the increased extract yield (80%–87%). Also, the improvement in percentage yield of aqueous extract was determined with increasing dosage of all enzymes. To conclude, EAE significantly improved the recovery of total carotenoids in the aqueous extract [23]. The authors also paid attention to the optimization of the most appropriate extraction conditions to carry out said EAE. Analyses performed by Wang et al. [46] showed that pH, extraction time, extraction temperature, and enzyme concentration all have significant effects on the enzymatic carotenoid extraction method from *Cordyceps militaris* (fungi, Ascomycota) by evaluating the antioxidant activities of the extract [46]. The scientific data also address the undesirable oxidation of carotenoids during standard solvent-based extraction and present the EAE as the better alternative for carotenoids high yield extraction. Strati et al. [120] demonstrated an increased recovery of carotenoids from tomato processing waste with the use of enzymes with pectinolytic and cellulolytic activities. They proposed EAE as a pre-treatment procedure before the solvent extraction which caused an increase in the extraction yields of analytes from plant sources [120]. Also, the drying step of the extraction could be omitted while EAE is implemented in the experimental platform. The isolation of carotenoids together with other biologically relevant compounds was optimized, too.

Mai et al. [121] presented research where the main intent was to investigate the performance of a combination of several enzymes: cellulase, pectinase, protease, and α-amylase in the extraction of oil rich in carotenoids from garlic [121]. The acquired data showed that the total carotenoid content increases linearly with the oil recovery, while EAE- based method was enacted. Nevertheless, they also pointed out that the high required ratio of the enzyme limits the economic potential. Therefore, further improvements should be made such as the optimization of combined extraction approaches where enzymatic degradation is performed together with the microwave or ultrasonic extraction [121].

### 4.2. EAE of Carotenoids from Daucus Carota L.

Carrot tissue EAE was presented in the literature some time ago. The data revealed that treatment with a combination of pectinase, cellulase, cellobiase, and pectin lyase increased the lycopene yield by approx. 50%. Enzyme treatments lead to changes in the microstructure and extractability, while some of the carotene complexes and interactions were broken. The EAE carrot sample pretreatment resulted in increased carotene extractability. Moreover, scientists, especially those wishing to follow the “green chemistry” expectations, tried to re-use the pomace remains after the production of the carrot juice. It is commonly used as feed or fertilizer, but it could also be a valuable source of carotenes. Thus, it is of utmost importance to present novel, “green chemistry” methods aimed at recovering the carotene-rich functional food ingredient from carrot pomace. During the extraction fine grinding of the suspension of carrot pomace in water using a colloid mill and the subjection of the pomace to enzymic hydrolysis, homogenization and concentration should be carried out. For the enzymic hydrolysis, Pectinex^®^ Ultra SP-L (PU; pectinase with hemicellulolytic activities, St. Louis, MO, USA) could be combined with either Cellubrix^®^ L or Cytolase CL (CE and CY, respectively; cellulolytic activity, St. Louis, MO, USA). The data showed that combinations of PU and CY was a more effective strategy than each of the enzyme alone and a 1:1 ratio was the most effective [122]. The co-extraction of carotenoids with other relevant compounds (such as pectins) could also be mentioned. This approach could add value to the additive for functional food formulation. In the recent study performed by Encalada et al. [123] the combination of EAE with ultrasound extraction was examined. It was determined that this combined extraction leads to an increased yield of extraction of pectins, α- and β-carotens, among others and hemicellulase was the enzyme that produced the highest increase in the yield of extraction [123].

In recent years, many researchers have focused on the extraction of analyte from carrots and other vegetables by using enzymatic treatments. Usually, manual methods of carotenoids extraction could cause undesirable flavor and color changes during the cell wall disintegration to release carotenoids. EAE has been found to prevent these negative changes during carotenoid extraction, the reason being that the extracted carotenoids by enzymes are still bound to proteins, so they provide stability to the color and structure of unsaturated pigment [25]. The utilization of enzymes mixture generated from microorganisms could also reduce the processing time for carotenoids extraction [25]. Around 50% of carotenoids can be lost during the extraction process, thus the use of enzymatic application prior to solvent extraction can efficiently reduce the extraction losses [124]. Applying enzymatic pre-treatments to the vegetable matrix can increase the cell wall permeability, enhancing the leaching process of carotenoids. Thus, a positive correlation between carotenoid extraction and enzyme pretreatment was found [125]. Holanda [126] obtained two times more carotenoid extraction after enzymatic hydrolysis of shrimp waste as compared to solvent extraction using oil. During solvent extraction, the carotenoid recovery increased to 17% to 31% after using alcalase pretreatment. Several studies have found that complex vegetable matrices could be hydrolyzed by using a combination of different enzymes [25] The application of raw enzymes has several benefits compared to the commercial enzymes due to their lower cost and shorter processing time in carotenoid extraction [6,25]. Water is also an important parameter to consider during EAE of carotenoids from vegetable matrices, as optimum water concentration is necessary for enzymatic hydrolysis of the cell wall matrix [124]. Agitation during enzymatic extraction facilitates the enzyme diffusion from the liquid phase into the vegetable matrix (solid phase). This rapid enzymatic adsorption can accelerate the lysis of the cell wall, leading to an increase in the extraction yield. Therefore, agitation plays an important role during the enzymatic extraction of carotenoids [61].

Industrially, many microorganisms are employed for the generation of various cellulolytic enzymes [127]. *Aspergillus niger* is one of the most important multi-enzymes producer microorganism to generate pectinases, hemi-cellulases, cellulases, glucoamylases, showing enzyme activities of 7.62, 15.86, 0.99 and 13.37 U mg^−1^ of protein respectively [15]. Cellulase and pectinase enzymes are commonly used during pretreatment before solvent extraction. Cellulase usually acts on the cellulose that is present below the first half layer of the cell wall in plants [14]. Cellulases can be produced from the reaction of cellulolytic microorganisms to domestic and agro-industrial waste. Currently, *Aspergillus niger*, *Trichoderma longibrachiatum,* and *Saccharomyces cerevisiae* are commonly used for cellulose production [128]. Pectin is also a major constituent of the cell walls in plants. Pectin is a polysaccharide composed of long chains of galacturonic acid with 1.4-α-links [129]. Pectinase enzymes can break down pectin compounds to release carotenoids [14]. The combination of different enzymes such as methylesterases and depolymerases is required to degrade galacturonate units in pectin compounds 38. The effect of EAE on carotenoids from Gac fruit (*Momordica cochinchinensis* Spreng.) was studied by Kha et al. [47]. The results showed that the highest extraction of the carotenoids content, specially β-carotene was achieved by using enzyme concentration at 0.1% (*w*/*w*) for pretreatment Kha et al. [47]. Strati et al. [120] also evaluated the use of cellulase and pectinase enzymes to assist the high-pressure extraction of carotenoids from tomato waste. It was concluded that the extraction yields of total carotenoid and lycopene were enhanced after the use of enzyme pretreatment before extraction.

## 5. Conclusions

The isolation of biologically relevant compounds from natural sources gets more attention nowadays since the quality of food is decreasing due to pollution affected by air, water, and climate changes. In the presented review, the advantages of carotenoids extraction from carrots with the use of two “green chemistry” methods, SC-CO_2_ and EAE, have been pointed out. The main benefits of using SC-CO_2_ for isolation of carotenoids are solvent-free products, no co-products, and low temperatures in the separation process. SC-CO_2_ is non-carcinogenic, non-toxic, non-mutagenic, non-flammable, and thermodynamically stable and CO_2_ does not usually cause oxidization of the analytes that are very important for the carotenoids. On the other side, EAE offers a faster extraction, higher recovery, reduced solvent usage, and lower energy consumption when compared to non-enzymatic methods. For the carotenoid’s extraction from carrots, EAE improves the extraction yields by disrupting the cell walls of the carrot’s tissues. The SC-CO_2_ method requires important technological investments, while EAE is a more convenient solution for the application in developing countries [121,130]. As presented above, both techniques offer important advantages over organic solvent and mechanically supported extractions and should be considered for the application instead of other methods for carotenoids extraction from *Daucus carota* L.

## Figures and Tables

**Table 1:** Comparison of some of the methods’ parameters for extraction with organic solvent, carbon dioxide extraction, and enzyme-assisted extraction (EAE).

Methods’ Parameters	Extraction with Organic Solvent	CO_2_ Extraction	EAE
Extraction yield	High	High	Low
Cost of the analysis	Medium	High	Low
Cost of the solvents	High	Low	Low
Ecological safety	Low	High	High
Time	Rapid	Rapid	Medium
Trace solvent residues in final product	Yes	No	No
Handling	Difficult	Difficult	Simple

**Table 2:** Concentrations and applications of different carotenoids from carrot.

Carotenoid	Concentrations [mg/g]	Extraction Methods		References	
**Total carotenoids**	0.16–0.38	10 g extracted with n-hexane/ethanol 96% (1:1, *v*/*v*) until colorless; kept at 20 °C and analyzed within 72 h		[51,52]	
**Carotenes (Hydrophobic Carotenoids)**		**Color**	**Application**	**Activities**	**References**
**β-carotene**	0.046–0.10	Orange	Nutraceutical; cosmetic; animal feed industries	Antioxidant, anticancer, precursor of vitamin A	[52]
**α-carotene**	0.046	Red	Nutraceutical and functional nutrients	Antioxidant, counteract heart disease and cancer	[53]
**Xanthophylls (Hydrophilic Carotenoids)**		**Color**	**Application**	**Activities**	**References**
**Lutein**	0.0011–0.0056	Golden Yellow	Poultry feed; functional nutrient	Antioxidant	[54]
**Zeaxanthin**	0.031	Yellow	Poultry and fish	Eye disease, Age-related macular degeneration	[55]

**Table 3:** Extraction of carotenoids from different sources by using supercritical extraction method.

Sample	Analyte	Conditions	References
Buriti (*Mauritia flexuosa*) pulp	β-carotene	20 MPa (pressure), 40 °C (temperature)	[98]
Microalgae *Chlorella vulgaris* (Chlorophyta)	carotenoids: canthaxanthin and astaxanthin	10 to 35 MPa (pressure) 40 and 60 °C (temperature)	[99,100]
Marine microalgae *Microchloropsis gaditana* (formerly *Nannochloropsis gaditana,* Ochrophyta, Eustigmatophyceae)	carotenoids and chlorophyll	10 to 50 MPa (pressure 40 and 60° C (temperature)	[101]
Cyanobacterium *Synechococcus* sp.,	carotenoids and chlorophyll	10 to 50 MPa (pressure) from 40 to 60 °C (temperature)	[102]
Rose fruit (*Rosa canina*)	carotenoids: lycopene, the β-carotene and lutein	15 to 45 MPa (pressure) from 40 to 80 °C (temperature) CO_2_ flow rate from 2 to 4 mL/min	[103]
Pitanga (*Eugenia uniflora* L.).	carotenoids: lycopene and rubixanthin	10, 15, 20, 25, 30, 35, and 40 MPa (seven levels of pressure) 40 and 60 °C (temperature)	[104]
Tomato Paste	lycopene and astaxanthin	pressure (from 10 to 42 MPa) temperature (40 to 60 °C)	[105]
Pink shrimp (*P. paulensis* and *P. brasiliensis*)	carotenoid components	from 10 to 30 MPa (pressure) 40 and 60 °C temperature	[62]
peach palm fruit (*Bactris gasipaes*)	total phenolic content, total flavonoids, total carotenoids	300 bar (pressure) 40 °C (temperature)	[106]
Gac fruit (*Momordica cochinchinensis Spreng.*) aril	lycopene, β-carotene	20 MPa (pressure) 50 °C (temperature)	[47]
Carrot	Total carotenoid content, α- and β-carotene, and lutein	27.6–55.1 MPa (pressure) from 40 to 70 °C (temperature)	[15]
Frozen watermelon	Lycopene	20.7–41.4 MPa (pressure) 70–90 °C (temperature)	[107]
Pumpkin (*Cucurbita maxima*)	Total carotenoid content, carotenoid profile	25 MPa (pressure) 50 and 80 °C (temperature)	[14]
